# Effects of Functionalized Nano-TiO_2_ on the Molecular Motion in Epoxy Resin-Based Nanocomposites

**DOI:** 10.3390/ma13010163

**Published:** 2020-01-01

**Authors:** Shihang Wang, Shihu Yu, Jianying Li, Shengtao Li

**Affiliations:** 1 State Key Laboratory of Electrical Insulation and Power Equipment, Xi’an Jiaotong University, Xi’an 710049, China; lijy@mail.xjtu.edu.cn; 2 Electric Power Research Institute of Guangdong Power Grid Corporation, Guangzhou 510080, China; yush0913@163.com

**Keywords:** epoxy resin, TiO_2_, nanocomposite, glass transition temperature, molecular motion

## Abstract

Epoxy resin-based nanocomposites have been widely researched for being potential insulating materials in high voltage power equipment. In this paper, nano-TiO_2_ particles were chosen and surface-modified by a silane coupling agent containing an epoxy group. The effect of functionalized nano-TiO_2_ doping on the physical properties of epoxy resin was studied. The results of differential scanning calorimetry show that *T*_g_ increased significantly and can be increased by up to 35 °C. Therefore, it is believed that the suppression of molecular motion by the addition of nanofillers works effectively in the case of this functionalized nano-TiO_2_ and a strong interaction between the epoxy resin and the nano-TiO_2_ was formed after surface modification. Consequently, dynamic mechanical properties, thermal conductivity, electrical conductivity, and trap characteristics of epoxy resin are all adjusted after introducing functionalized nano-TiO_2_. All of these physical properties were analyzed from the perspective of suppression of molecular motion, and it is of significance to establish the theory of a nanocomposite dielectric. Besides, the results show that the epoxy/TiO_2_ nanocomposite is expected to be applied in the insulation system of electrical equipment.

## 1. Introduction

The epoxy resin insulating system is widely used in high voltage insulators, transformers, cable terminations, bushings, power apparatus and so on. Epoxy resin-based nanocomposites used as a new generation of dielectric materials in power equipment have been extensively researched for more than ten years, and many modified physical properties have been obtained and analyzed from different aspects. However, we still need more detailed data and analysis to enrich the theoretical system of epoxy resin-based nanocomposites for a lot of fundamental questions have not been answered.

Glass transition temperature (*T*_g_) of an epoxy resin-based nanocomposite, the most basic parameter, is determined by the preparation process and surface modification of the doping nanoparticles. Based on reported results, the introduction of silane coupling agents-modified nano-SiO_2_ decrease the *T*_g_ of epoxy resin according to the loss factor of mechanical modulus [1]. Similar results of decreased *T*_g_ have been reported about nano-Al_2_O_3_ [2,3,4,5], nano-TiO_2_ [3,6], nano-ZnO [7], and nano-MgO [8] fillers. There have been similar reports of increased *T*_g_, although the same kind of nanofiller was used. As reported, *T*_g_ of epoxy resin was increased by 8 °C with the incorporation of 1 wt.% TiO_2_ nanoparticles surface modified with gallic acid esters [9]. The introduction of nano-SiO_2_ modified by poly(propylene glycol)bis(2-aminopropyl ether) can increase the *T*_g_ slightly, no more than 6 °C [10]. There are few reports indicating that a composite metal-oxide nanofiller has led to a significant increase in *T*_g_ of epoxy resins. In terms of engineering application, the existing methods to increase the *T*_g_ have limited effect. Above all, there is no consistent conclusion on the effect of metal-oxide nanofiller on the *T*_g_, and it is not very clear how metal-oxide nanofillers affect the movement of the molecular chains in epoxy resin. Besides, *T*_g_ is a parameter that reflects not only the glass transition process but also many related physical properties.

In this paper, epoxy/TiO_2_ nanocomposites were prepared with significantly improved *T*_g_. The effect of functionalized nano-TiO_2_ on the motion of molecular chains was analyzed. The properties related to molecular motion were investigated for a better understanding of the internal principle in an epoxy resin-based nanocomposite dielectric.

## 2. Materials and Methods

### 2.1. Materials

The studied epoxy resin was a bisphenol-A resin (E51). As a curing agent methyl, tetrahydrophthalic anhydride hardener (MTHPA) was used at 80 phr. Tris (dimethylaminomethyl) phenol (DMP-30) was used as an accelerator at 1.5 phr. For nano-fillers, highly pure grades of commercial particles of rutile titanium dioxide (TiO_2_, the average diameter of 25 nm) were added by the weight ratio. γ-(2,3-epoxypropoxy) propytrimethoxy silane (KH560) was used as a coupling agent for surface modification of TiO_2_ particles. The samples prepared were unfilled sheets of epoxy resin and four kinds of epoxy/nanocomposites which contain 0.1 wt.%, 1 wt.%, 3 wt.% and 5 wt.% addition of TiO_2_ nanoparticles.

### 2.2. Fabrication of Epoxy Nanocomposites

**Figure 1:**
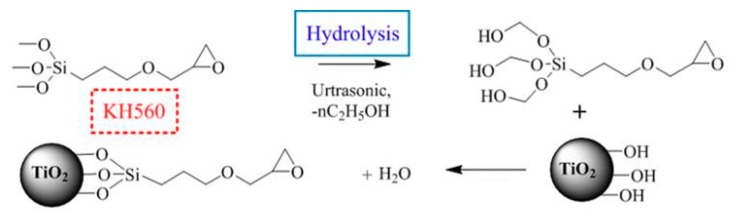
Reaction of surface functionalization of nano-TiO_2_ and epoxy curing.

Nanoparticles need to be well dispersed and distributed in a matrix for better unleashing potential. To reduce the agglomeration and improve the good adhesion between fillers and matrix, the surface functionalization of nano-TiO_2_ was performed by a solution mixing method. A moderate amount of nano-TiO_2_ particles was firstly dispersed in ethanol and then the mixed solution was dispersed using a homogeniser instrument (Stansted Fluid Power LTD., Essex, UK). A Silane coupling agent (KH560) having a mass ratio of 1.5% to the nanoparticles was added to the mixed solution of ethanol and water. Then, two solutions were mixed in a beaker and subjected to ultrasonic vibration for 10 min. Alkoxy group of silane was hydrolyzed to produce silanol, which was connected on the surface of nanoparticles, as shown in Figure 1.

Before the process of preparing epoxy resin specimens, epoxide was pre-heated to 60 °C in an oven to reduce its viscosity then mixed with the epoxy resin in a three-necked, round-bottomed flask. Heating during the stirring process allowed the nanoparticles to be uniformly mixed with the epoxy and evaporated the solvent. The hardener was added into the epoxy/nano-filler solutions and then the mixture was stirred for 15 min. This step was accomplished in a vacuum chamber to remove the gas bubbles. Next, the mixture was poured into the mold and placed into the oven at 80 °C to cure for 2 h, 105 °C to cure for 2 h followed by 4 h at 120 °C.

During the curing process, the mixing functionalized nano-TiO_2_ particles were connected to the molecular chain of epoxy resin through the crosslinking reactions of epoxy groups. In epoxy/TiO_2_ nanocomposites, tight reticulate structures connecting epoxy, silane coupling agent, nano-TiO_2_ and curing agent were formed though a reaction. Neat epoxy as well as functionalized epoxy/TiO_2_ nanocomposites with weight proportion of 0.1%, 0.5%, 1%, 3% and 5% were prepared. Before experiments, all samples were vacuum dried at 80 °C for 48 h.

### 2.3. Characterization

**Figure 2:**
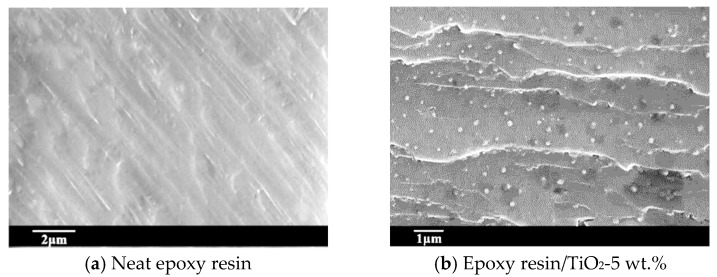
Micrograph of fracture surface of neat epoxy resin and its nanocomposite. (**a**) Neat epoxy resin; (**b**) Epoxy resin/TiO_2_-5 wt.%.

A scanning electron microscope (Keyence, Osaka, Japan) was used to verify the uniform dispersion of the nano-TiO_2_ in the samples. Samples were broken in liquid nitrogen and sputter-coated with gold, and the fracture surface was showed in Figure 2. It can be observed that the fracture surface of neat epoxy resin shows no impurity particles. The fracture surface of the epoxy/TiO_2_ sample with 5 wt.% presents spherical particles. The nanoparticles were uniformly distributed without the introduction of voids and other defects. From the perspective of flatness, the fracture surface of neat epoxy resin was obviously smoother compared with the nanocomposite sample. For the influence of nano-TiO_2_ to the mechanical properties of the epoxy resin matrix, the fracture surface represented beach stripes like crack.

*T*_g_ was measured using Differential Scanning Calorimetry (Mettler Toledo, Zurich, Switzerland). The samples were heated from 20 °C to 200 °C at a rate of 10 °C per minute under nitrogen atmosphere. This cycle was repeated twice for each sample and the second cycle was considered for calculation.

Changes in the viscoelastic properties of the samples were determined by Dynamic mechanical analysis using a DMA 861 analyzer (Mettler Toledo, Zurich, Switzerland). Briefly, samples were subjected to oscillating loading (Force was 3 N and frequency was 1 Hz) under bend load from −90 °C to temperature higher than the peak temperature of the transition (Rate of temperature rise was 3 °C/min).

Thermal conductivity was measured by the laser flash diffusivity instrument (NETZSCH LFA447, Bavaria, Germany) at different temperatures. The samples were 1 mm × 10 mm × 10 mm with a graphite coating surface.

Absorption currents under dc voltage were measured using electrometer (Keithley 6517B, Johnston, IA, USA) and three-electrode system. For the purpose of electrical measurements, gold electrodes were deposited on the sample surface to form additional electrodes. The dc voltage was 500 V and the samples were with the thickness of 1.5 mm.

Surface potential decay (SPD) curves of the samples after charged under dc voltage were obtained to calculate the surface trap parameters [11,12]. Samples with a thickness of 0.5 mm were charged by a needle-mesh electrode (needle electrode and mesh grid were applied with −8 kV and −4 kV) for 3 min, before quickly moved to the Kelvin probe of an electrostatic voltmeter(Trek, New York, NY, USA), and the surface potential was recorded [11].

## 3. Results

### 3.1. Glass Transition Temperature

**Figure 3:**
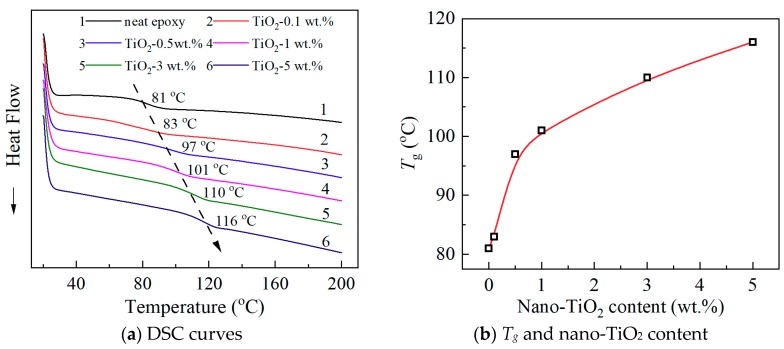
DSC curves and *T_g_* of neat epoxy and epoxy/TiO_2_ nanocomposites. (**a**) DSC curves; (**b**) *T_g_* and nano-TiO_2_ content.

Figure 3a shows DSC spectra. The glass transition process was clear in the temperature range between 80 and 120 °C. In Figure 3a, a black arrow was drawn as a guide for the *T*_g_ variation. The *T*_g_ increases with the increasing nano-TiO_2_ content, and it can be estimated to be 81 °C for the neat epoxy sample, and 116 °C, with a remarkable increment, for the sample with 5 wt.% nano-TiO_2_. The increment was up to 35 °C. Figure 3b gives the relation between *T*_g_ and nano-TiO_2_ content. *T*_g_ is directly depending on the property of molecular motion. It obviously indicates that the molecular chain of the nanocomposites needs a higher temperature to undergo the glass transition process, which means that the movement of the molecular chains is inhibited by the doping of functionalized nano-TiO_2_. As mentioned above, it is meaningful that insulating material used epoxy resin has a relatively high *T*_g_. Compared with the similar reports of nanocomposites, the increment of *T*_g_ in this paper is prominent and needs more research. Thus, molecular motion in epoxy/TiO_2_ nanocomposites and its effects were analyzed from the perspective of several physical properties.

### 3.2. Dynamic Mechanical Analysis

**Figure 4:**
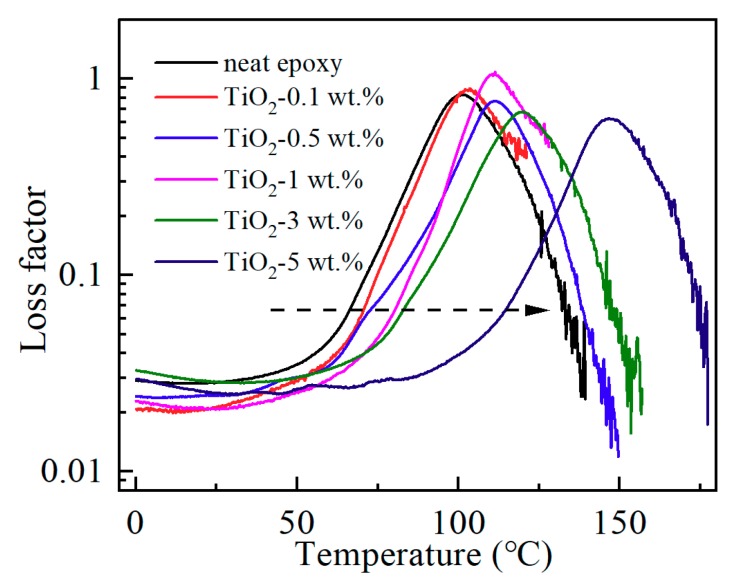
Loss factor of epoxy/TiO_2_ samples.

Dynamic mechanical analysis of neat epoxy resin and its nanocomposites at different filler loadings have been carried out in this study to investigate the variation of loss factor (tan δ), which is the ratio of loss modulus and storage modulus and a ratio of viscous to the elastic response. The value of tan δ can be used to analyze the physical property of the interface region in polymer-based nanocomposites. As shown in Figure 4, the peak value of the loss factor gives no obvious change indicating similar viscous behavior. Therefore, damping properties of epoxy resin were not changed, which means the nano-TiO_2_ filler and the matrix resin are well bonded together, and an interface between the nano-TiO_2_ filler and the matrix resin is ideal, with no apparent voids and other defects. As shown in Figure 4, the peak temperature of the loss factor increases significantly with the increasing nanofiller content. The right shift of the curves tan δ–T indicated not only the increase of *T*_g_ but also the improvement of the crosslinking degree of the materials. By the way, the *T*_g_ obtained from the results of dynamic mechanical analysis is different from the DSC results. This is mainly due to the different principles of different test methods, and in addition, the test parameters including the heating rate are also different.

### 3.3. Thermal Conductivity

**Figure 5:**
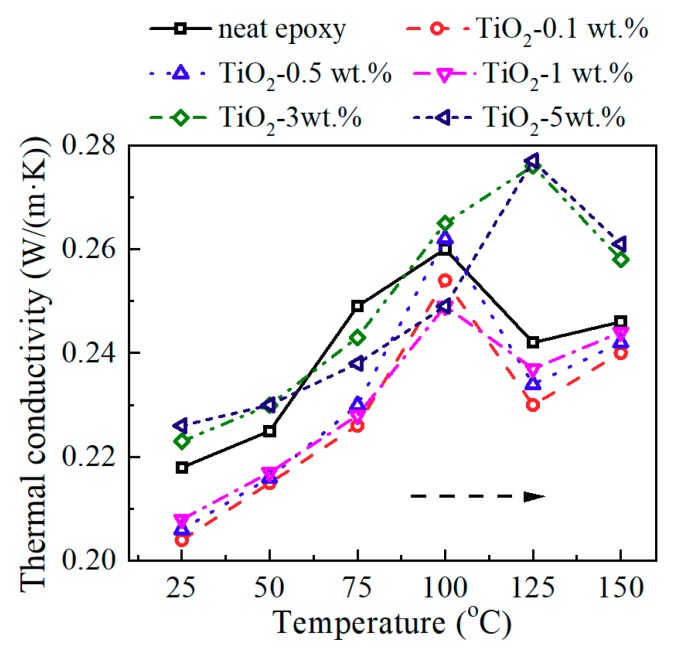
Thermal conductivity of epoxy/TiO_2_ samples at different temperature.

Figure 5 shows the thermal conductivity of neat epoxy and epoxy/TiO_2_ nanocomposites at different temperatures. A distinct broad peak appears at the *T*_g_. This peak was caused by the leap of heat capacity during the glass transition process. As mentioned above, nano-TiO_2_ introduction obviously increased the *T*_g_, thus the broad peak shifted to the high temperature.

**Figure 6:**
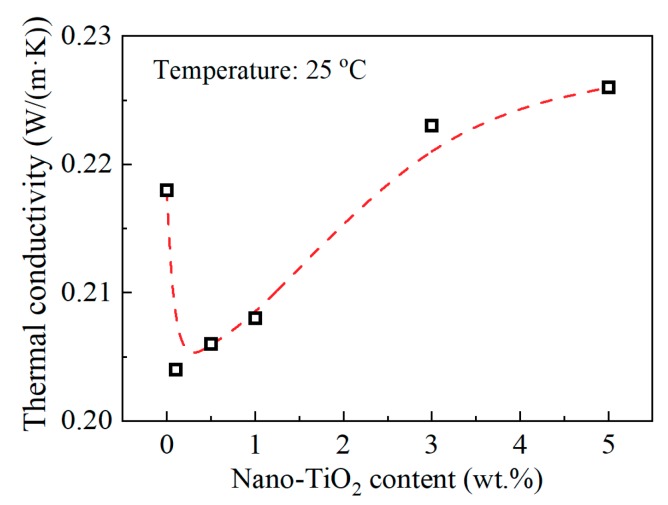
Thermal conductivity and nano-TiO_2_ content at 25 °C.

Figure 6 shows the relation between thermal conductivity and nanofiller loading content at 25 °C. The thermal conductivity decreased after introducing nano-TiO_2_, and then it increased with the increasing nano-TiO_2_ content. The thermal conductivity of the composite depends mainly on the thermal conductivity of the filler and the epoxy resin matrix, the content, and distribution of the filler, as well as the interface between filler and matrix. Since the filler and the matrix are two phases with different thermal conductivities, respectively, this will lead to scattering phenomena of phonons that transmit thermal vibrations, caused by factors such as reflection, refraction, interference, and retardation. These phenomena are like the scattering phenomena of vibration and waves at the two-phase interface with different elastic coefficients. At the same filler content, the fillers with smaller particle sizes have larger specific surface areas. Thus, in composites with nano-sized particles, the phonon scattering at the thermal conduction path will be more severe for more two-phase interface. Therefore, results in Figure 6 can be interpreted in two parts. At relatively low nanofiller content, 0.1 to 1 wt.%, the thermal conductivity of the filler itself did not contribute much to the nanocomposites. Scattering phenomena of phonons at the epoxy/nano-TiO_2_ interface lead to the decrease of thermal conductivity. At relatively high nanofiller content, 3–5 wt.%, more TiO_2_ fillers lead to the slight increase of thermal conductivity, for the contribution of the nano-TiO_2_ filler itself with high thermal conductivity. Of course, it may also include the role of heat transfer channels brought about by nanofiller agglomeration at relatively high loading content. Note that at high doping content, the phonon scattering at the interface still exists. The variation of thermal conductivity is the result of these two factors.

### 3.4. Electric Conduction

**Figure 7:**
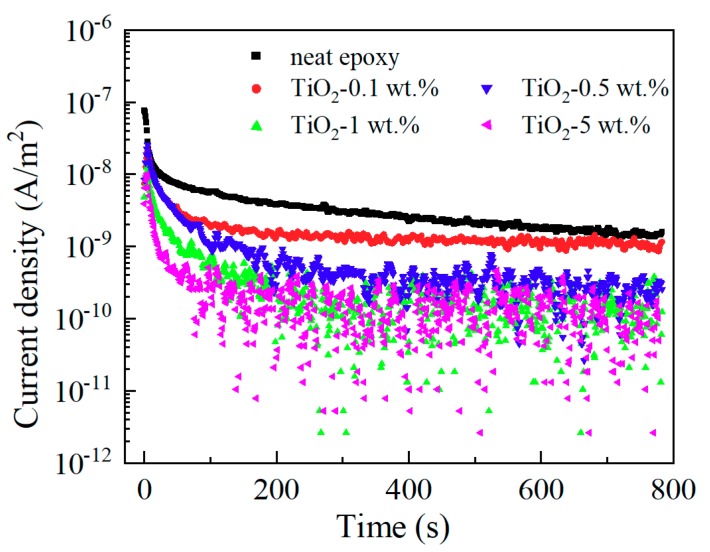
Absorption current of epoxy/TiO_2_ samples.

It is well known that the charging current decays with the time immediately after the application of a step-function voltage across an insulating material between two metallic electrodes, and this charging current is also called absorption current. Figure 7 presents the absorption currents of neat epoxy resin and its nanocomposite samples. The currents reach steady state at about 800 s. There are three possible mechanisms that may be responsible for this current decay phenomenon. These mechanisms are ionic conduction, dipolar polarization, and electronic conduction. Because the electric field of measurements is low, electronic conduction is very small and can be neglected here. Hence it is considered that the stable currents are mainly the contribution of ionic conduction. During the fabrication process of the sample in our work, the cleanliness was guaranteed, and a neat epoxy resin sample went through the same fabrication process as the nanocomposite samples besides the introduction of nanofiller. Therefore, the content of impurity in neat epoxy resin and its nanocomposites has no significant difference, which means ionic charge carrier content has no big difference in each sample. The ionic conductivity is related to density of charge carrier and charge mobility.

**Figure 8:**
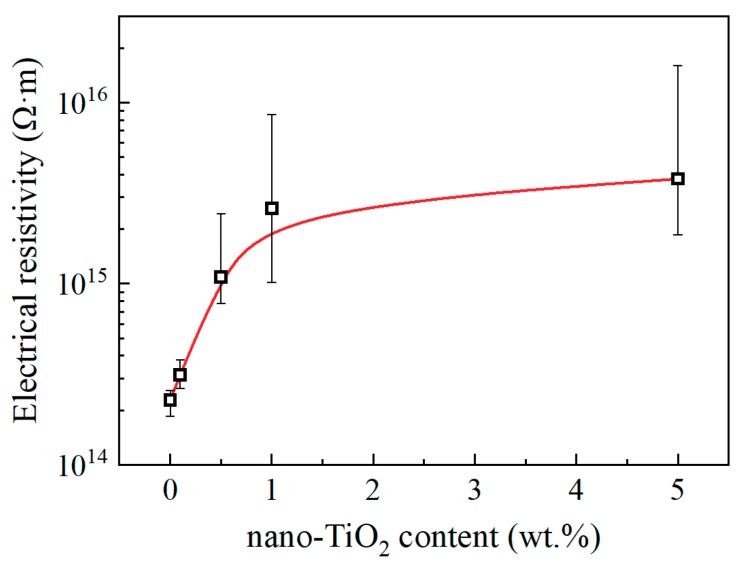
Electrical resistivity and nano-TiO_2_ content.

The steady value of absorption current decreased with the increasing nano-TiO_2_ content, and the electric resistivities calculated by them are shown in Figure 8. Consequently, we draw the conclusion that functionalized nano-TiO_2_ doping decreases the charge mobility and suppresses ionic conduction of epoxy resin. This result is also related to the restricted movement of molecular chains.

### 3.5. Charge Trap

**Figure 9:**
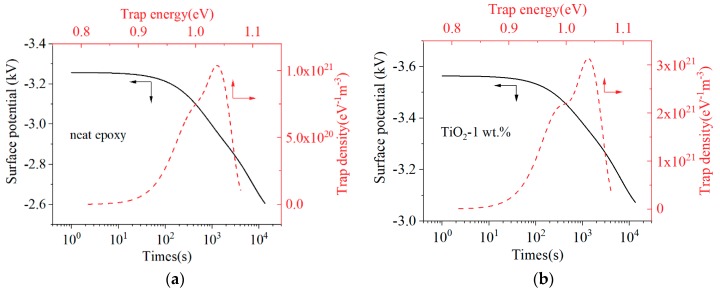
Surface potential decay curves and trap characteristics: (**a**) Neat epoxy; (**b**) Epoxy resin/TiO_2_-1 wt.%.

The charge trap characteristic is an important property for polymer insulating materials, and it reflects the effect of molecular motion and defects on the transport of charge carriers under an electric field. Different charge trap characteristics will modulate different insulating properties [13,14]. By comparing the surface potential of the epoxy sample and TiO_2_-1 wt.% sample, it illustrates that nano-TiO_2_ doping decreased the speed of surface potential decay. According to the theory of Simmons, the trap level and trap charge density could be calculated [12,15,16]. As shown in Figure 9, the surface potential increases and its decay rate decreases after nano-TiO_2_ doping. The calculated trap energies of neat epoxy sample and TiO_2_-1 wt.% sample are mainly around 1 eV, which can be regarded as deep traps. The results show the nano-TiO_2_ doping to epoxy resin insulating materials increased the density of deep traps significantly.

## 4. Discussion

Based on these experimental results, it can be concluded that the introduction of functionalized nano-TiO_2_ will hinder the motion of molecular chains of epoxy resin insulating materials. As a result, the glass transition process of epoxy resin occurs at a higher temperature range and most related physical properties were regulated accordingly.

**Figure 10:**
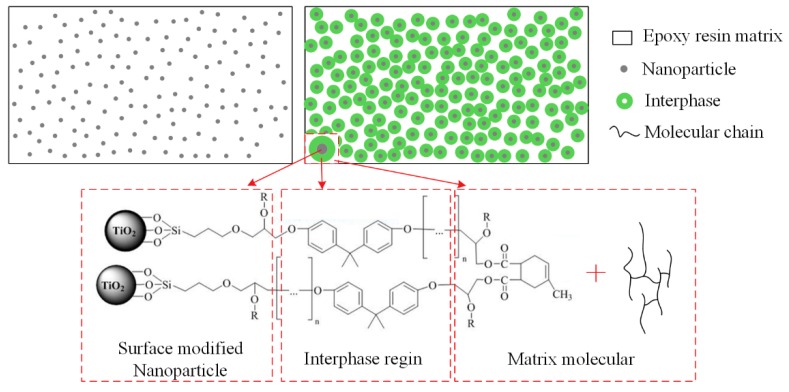
Absorption currents of epoxy/TiO_2_ samples.

First, it can be considered that the restriction of molecular chain movement is mainly related to the interphase, interfacial region of nanofiller and polymer matrix. Interphase can be considered as the third phase in epoxy/TiO_2_ nanocomposites. It possesses properties that are distinct from the matrix resin. As represented in Figure 10, although the volume ratio of the nanoparticles is very small in the matrix, the interphase with a thickness of tens of nanometers will have a relatively large volume ratio. Therefore, the physical properties of interphase will dominate the physical properties of the nanocomposites, and controlling the properties of interphase is the key to modulate the properties of nanocomposite dielectric. In this paper, interphase in epoxy/TiO_2_ nanocomposites also plays an important role. As shown in Figure 1, the use of a silane coupling agent containing an epoxy group functionalizes the surface of nano-TiO_2_ fillers. This allows the nano-TiO_2_ to be coated with epoxy groups, which can participate in the curing reaction of the epoxy resin. Consequently, the nano-TiO_2_ was bounded to the molecular backbone. As shown in Figure 10, there will be an interphase region with a certain thickness that affected by the connection effect to nanoparticles. The thermal motion of the molecular chains in the interphase region becomes difficult for the high mass of nano-TiO_2_. With the increasing nanofiller content, the volume ratio of the interphase region will increase continuously. It is generally considered agglomerations of nanofiller under relatively high doping content will bring defects and voids that will cause the thermal motion of the matrix molecular chain. However, this phenomenon did not occur within the study content ranging up to 5 wt.% in this paper.

The interphase region in epoxy/TiO_2_ nanocomposites represents a stronger structure with restricted molecular chains. The most obvious property of epoxy resin affected by the interphase region is the enhanced *T*_g_. It increases the maximum service temperature of epoxy resin insulating materials in power equipment. Besides, it will decrease the permittivity caused by displacement polarization of molecular chains. The thermal conductivity decreases at low doping content, but not large, which means just a little effect on the heat dissipation efficiency. For electric conduction at relatively low electric fields, it is mainly the impurity molecule, water molecule and other small molecules that are involved in charge transport. Some structures should provide channels, allowing ions space to move. Therefore, the motion of the molecular chain will affect the charge mobility. The inhibition of molecular chain motion will lead to a decrease of carrier mobility, and this is the reason why the *T*_g_ has a similar variation trend with the electric resistivity.

Some results reported in the literature usually show that inorganic fillers can improve the thermal conductivity of the composites to different degrees, and even a small amount of nano-sized fillers can improve the thermal conductivity slightly [17,18]. As stated in the introduction, there is no consistent conclusion on many physical properties of epoxy resin-based nanocomposite. Obviously, the results in this paper that thermal conductivity of epoxy/TiO_2_ nanocomposite decreased at low doping content, are novel and helpful to further understand the diversity of nanocomposite dielectric. It has been explained in the result section that the decrease of thermal conductivity is caused by the scattering phenomena of phonons at the epoxy/nano-TiO_2_ interface. Combining the concept of interphase, it may be speculated that the interphase has a different thermal conductivity comparing with the epoxy resin matrix. Under this assumption, there will be another interface between interphase and epoxy resin matrix, and phonon scattering at this interface may also be a reason for the reduction of thermal conductivity.

As to the *T*_g_, its value keeps increasing within the doping content range in this paper. It is not known to what extent the *T_g_* can be increased with continuously increasing the nano-TiO_2_ content. But it is certain that the high content of nanoparticles will degrade the insulation performance. For example, the variation trend of electric resistivity with increasing nanofiller content is not consistent with the variation trend of *T*_g_ at relatively high nano-TiO_2_ content. It is speculated to be caused by more impurities in samples with more nanoparticles, and electric resistivity may be significantly reduced if more nanoparticles are added. As to the breakdown strength, a very important parameter of insulating materials, it reached the maximum at a relatively low nanofiller content as reported [13,19,20]. Therefore, a wide range of physics properties of epoxy/TiO_2_ nanocomposites including maximum service temperature should all be considered to determine the best loading content of nano-TiO_2_ in the engineering application as high voltage insulating materials.

It is generally accepted that polymer based nanocomposites have some improved performances comparing with their original polymers. But such improved performances are usually explained in terms of each model or each thought corresponding to each of performances and phenomena. It is necessary to explain as many performances as possible based on one comprehensive model. Therefore, in our work, the effect of functionalized nano-TiO_2_ on the molecular motion of epoxy resin was studied according to the analysis of *T*_g_, loss factor of modulus, thermal conductivity, electric resistivity, and trap characteristics.

## 5. Conclusions

The work presented in this paper investigated epoxy/TiO_2_ nanocomposites with an enhanced glass transition temperature. Several conclusions were achieved.

(1) Nano-TiO_2_ surface modified by γ-(2,3-epoxypropoxy) propytrimethoxy silane can form a strong interaction with the epoxy resin matrix and moderate content of nano-TiO_2_ doping effectively adjust the property of epoxy resin.
(2) The *T*_g_ increased significantly after nano-TiO_2_ doping and it can be increased by up to 35 °C. The thermal motion of epoxy resin molecular was suppressed by the addition of functionalized nano-TiO_2_.
(3) Dynamic mechanical properties, thermal conductivity, electrical conductivity and trap characteristics of epoxy resin are all adjusted after nano-TiO_2_ doping. All of these physical properties of epoxy/TiO_2_ nanocomposite dielectric was related to the suppression of molecular motion.
